# Supplementary figures and images for: IRE1α regulates macrophage polarization, PD-L1 expression, and tumor survival
Source: PLoS Biol. 2020 Jun 10;18(6):e3000687. doi: 10.1371/journal.pbio.3000687 (PMC7307794; doi:10.1371/journal.pbio.3000687)

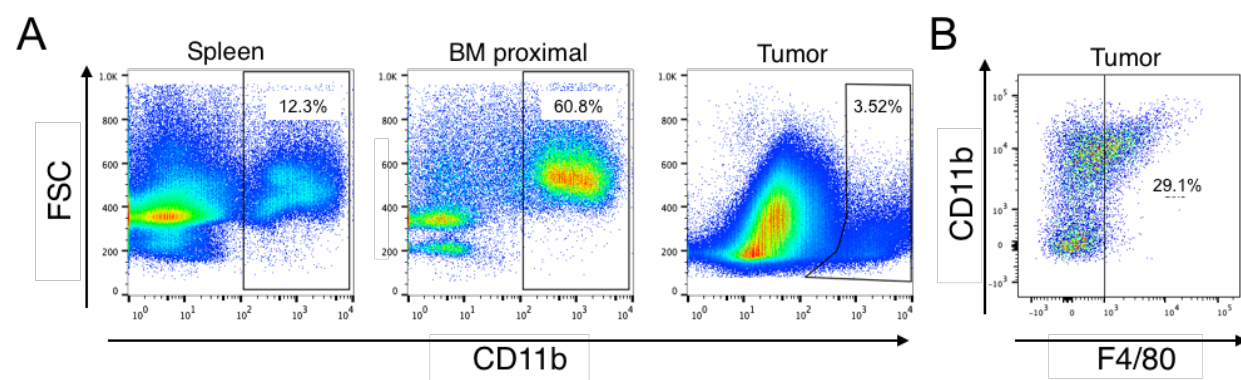

S1 Fig

Supplement: S1 Fig — (A). Flow cytometry analysis of CD11b+ cells in the spleen, the BM, and within the TME of B16.F10 tumors in C57BL/6 mice carrying the Xbp1-Venus fusion transgene. (B) Analysis of F4/80 expression on CD45+ cells in B16.F10 tumors. BM, bone marrow proximal to the tumor; TME, tumor microenvironment. (PDF) [file pbio.3000687.s001.pdf]

**A**

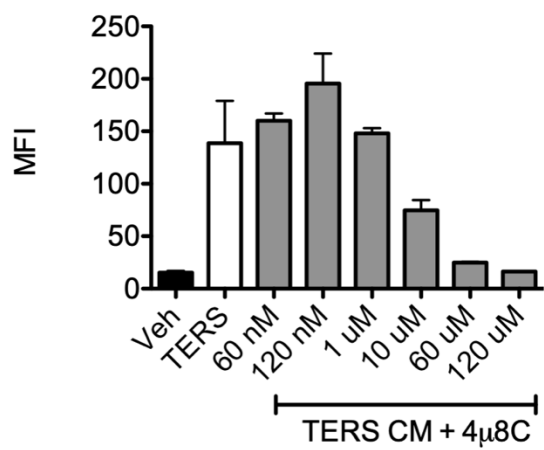

**B**

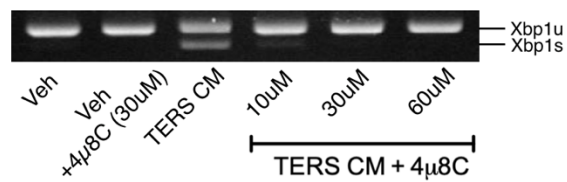

**S2 Fig**

Supplement: S2 Fig — Dose-dependent 4μ8C-mediated inhibition of ERAI induction by TERS CM in B16.F10 melanoma cells (A) and quantification of 4μ8C inhibition of Xbp1 splicing in C57BL/6 mice macrophages stimulated with TERS CM (B). Data are included in S2 Data. ERAI, ER stress-activated indicator; TERS CM, transmissible ER stress conditioned medium; XBP1, X-box binding protein 1. (PDF) [file pbio.3000687.s002.pdf]

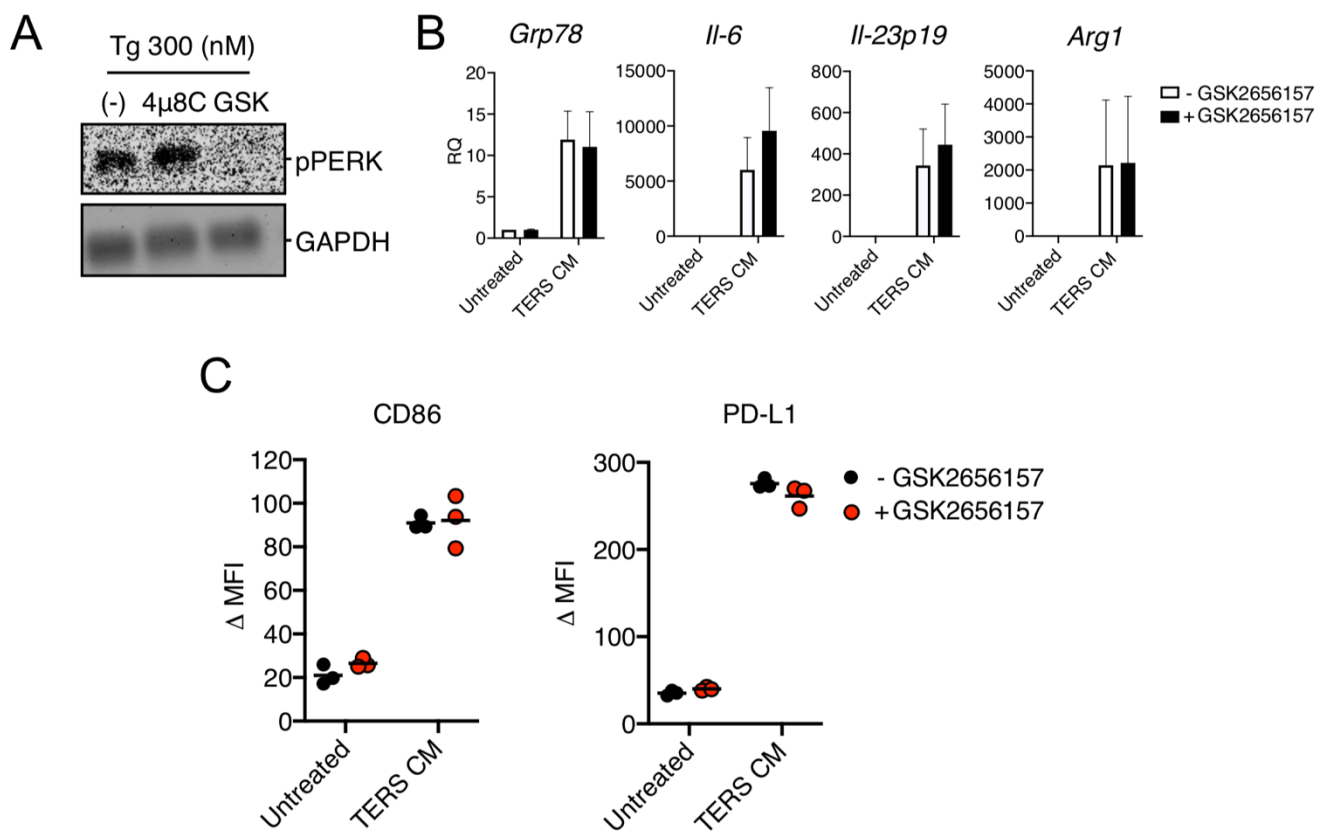

S3 Fig

Supplement: S3 Fig — (A) Western blot analysis for pPERK in whole-cell lysates from BMDM treated with Tg with or without 4μ8C (30 μM) or GSK2656156 (10 nM). (B) Expression of selected genes by RT-qPCR by mRNA from BMDM cultured in TERS CM or in vehicle Veh CM with or without GSK2656157 (50 nM) (n = 4). Error bars represent SEM. (C) Surface expression (flow cytometry) of CD86 and PD-L1 in BMDM cultured in TERS CM or in vehicle Veh CM with or without GSK2656157 (50 nM). Data are included in S2 Data. BMDM, bone marrow–derived macrophage; CM, conditioned medium; IIS, proinflammatory/immune-suppressive; PD-L1, programmed death ligand 1; PERK, PKR-like ER kinase; pPERK, phosphorylated PERK; RT-qPCR, reverse transcriptase quantitative PCR; TERS CM, transmissible ER stress CM; Tg, thapsigargin. (PDF) [file pbio.3000687.s003.pdf]

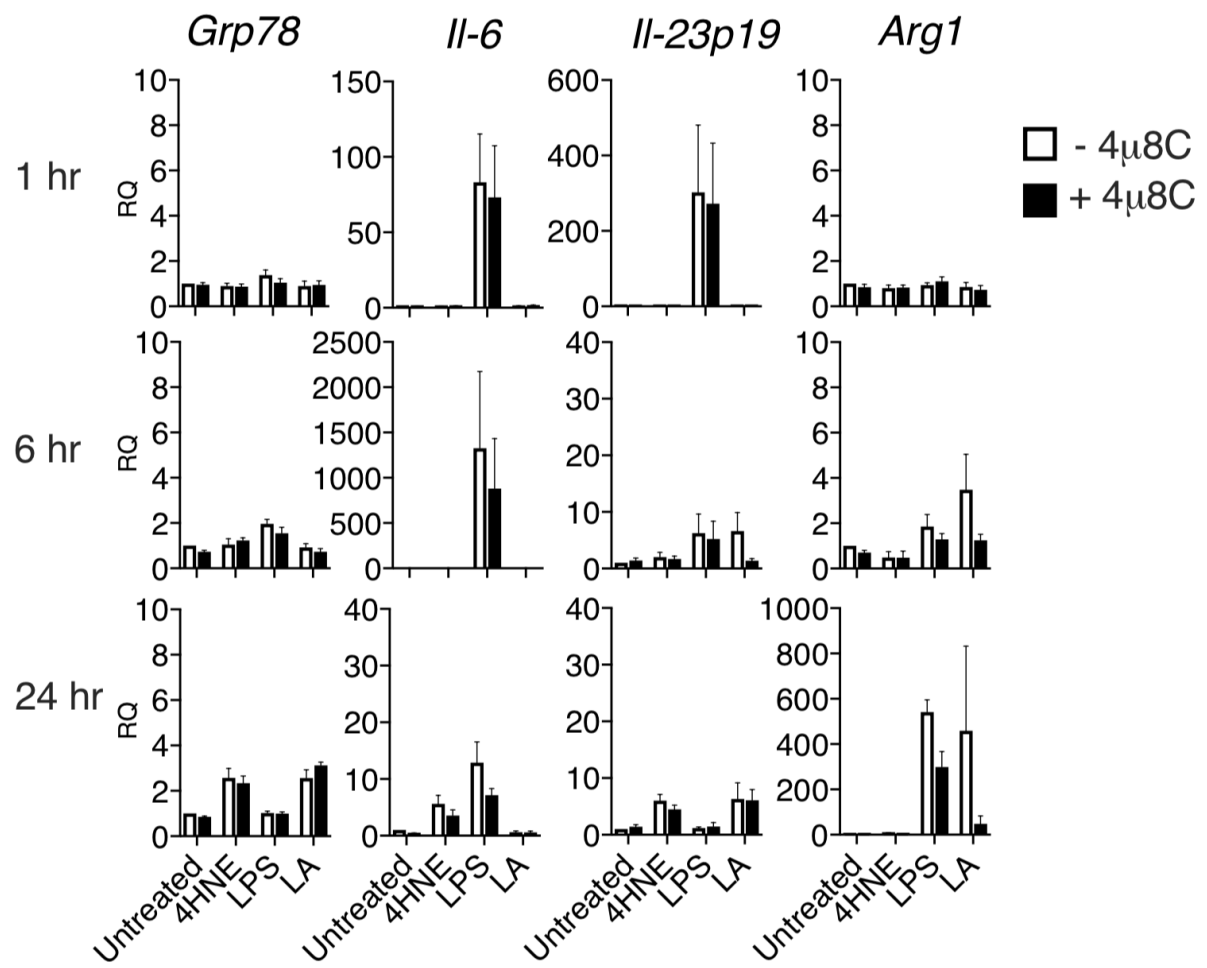

**S4 Fig**

Supplement: S4 Fig — At the indicated time points, RNA was isolated using Nucleospin 2 kit and processed for RT-qPCR. Values represent the mean ± SEM (n = 5 per group). Data are included in S2 Data. 4HNE, 4-hydroxynonenal; BMDM, bone marrow–derived macrophage; LPS, lipopolysaccharides; RT-qPCR, reverse transcriptase quantitative PCR. (PDF) [file pbio.3000687.s004.pdf]

**A**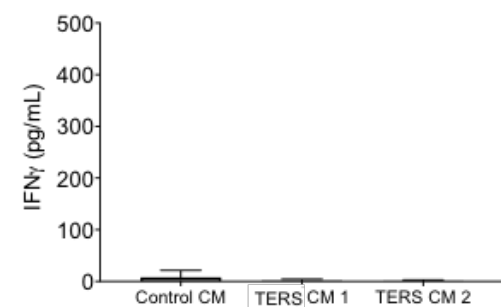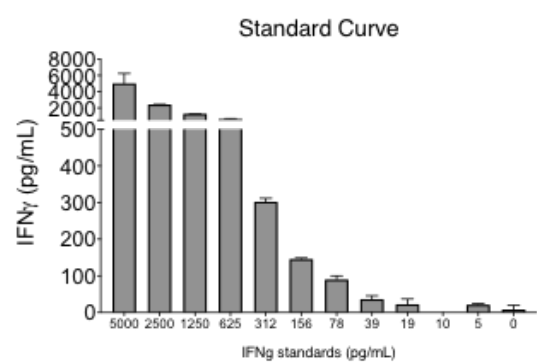**B**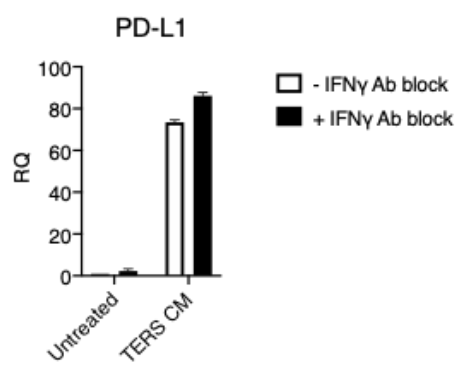**C**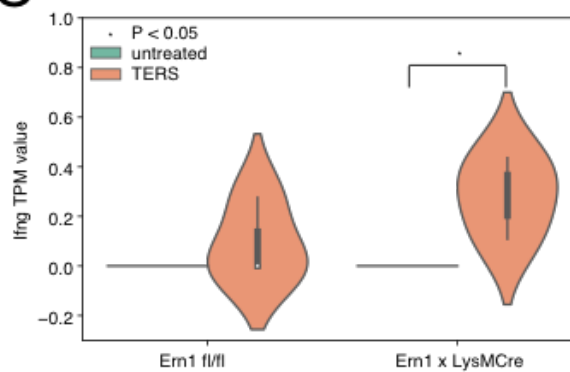**S6 Fig**

Supplement: S6 Fig — The standard curve provided by the manufacturer was used to quantify each sample (A). BMDMs generated from wild-type C57BL/6 mice were untreated or treated with TERS, with and without a blocking antibody for IFNγ for 18 hours. RNA was isolated using Nucleospin 2 kit and processed for RT-qPCR (B). Boxplot showing the IFNG gene expression in Ern1(fl/fl) and Ern1 LysMCre groups from the RNASeq data set (C). Data are included in S2 Data. BMDM, bone marrow–derived macrophage; CM, conditioned medium; IFNγ, interferon gamma; RNASeq, RNA sequencing; RT-qPCR, reverse transcriptase quantitative PCR; TERS CM, transmissible ER stress CM. (PDF) [file pbio.3000687.s006.pdf]

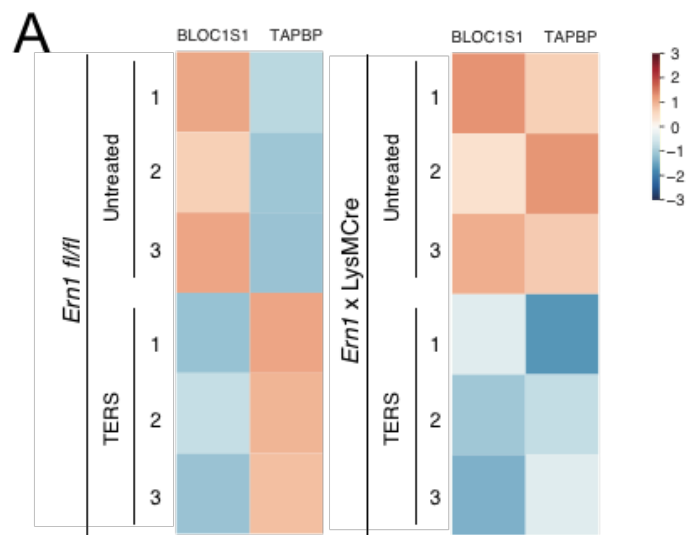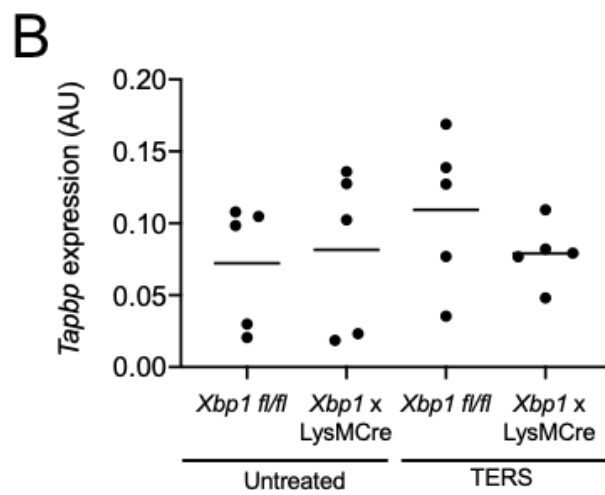

**S7 Fig**

Supplement: S7 Fig — RNASeq analysis of Tapbp expression in untreated or TERS CM–treated wild type and Ern1-CKO BMDM (A). RT-qPCR analysis of Tapbp expression analysis in untreated or TERS CM–treated wild type and Xbp1-CKO BMDM (B). Data are included in S2 Data. BMDM, bone marrow–derived macrophage; CKO, conditional knock-out; RNASeq, RNA sequencing; Tapbp, tapasin; RT-qPCR, reverse transcriptase quantitative PCR; TERS CM, transmissible ER stress conditioned medium; Xbp1, X-box binding protein 1. (PDF) [file pbio.3000687.s007.pdf]

**A**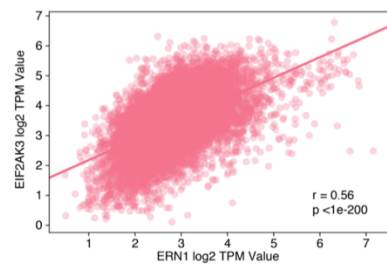**B**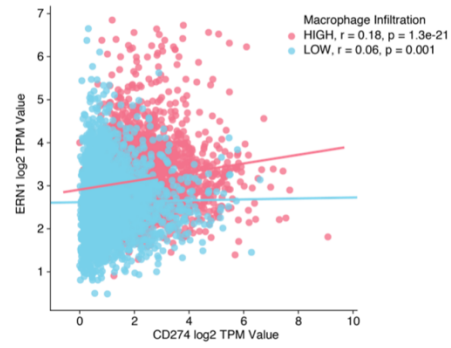**C**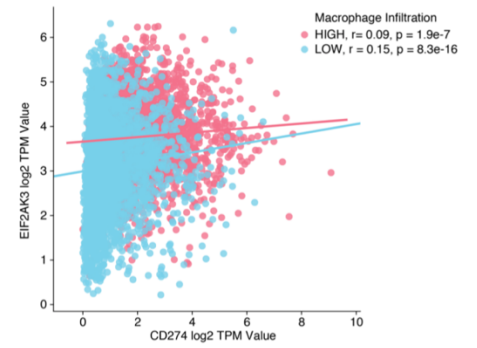**S8 Fig**

Supplement: S8 Fig — (A) Spearman correlation between ERN1 expression and EIF2AK3 expression from TCGA pancancer study (n = 9,607). Both genes are normalized to TPM and in log2 scale. (B) Spearman correlation between ERN1 expression and CD274 expression from TCGA pancancer study (n = 9,607). Red dots are samples with high macrophage infiltration scores (>70%), and blue dots are samples with low macrophage infiltration scores (<30%). (C) Spearman correlation between EIF2AK3 expression and CD274 expression from TCGA pancancer study (n = 9,607). Red dots are samples with high macrophage infiltration scores (>70%), and blue dots are samples with low macrophage infiltration scores (<30%). Data are included in S2 Data. EIF2AK3, translation initiation factor 2; TCGA, The Cancer Genome Atlas; TPM, transcripts per million. (PDF) [file pbio.3000687.s008.pdf]

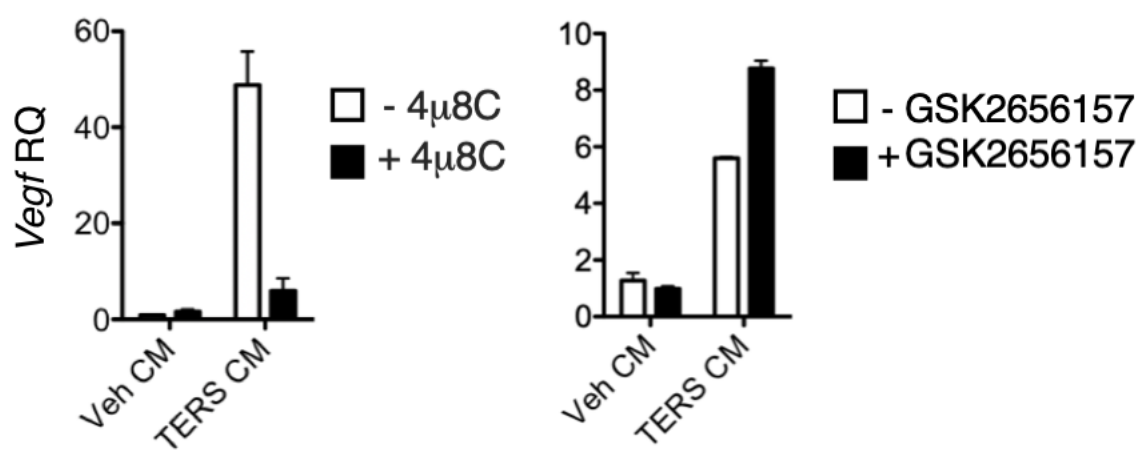

**S10 Fig**

Supplement: S10 Fig — Expression of Vegf by RT-qPCR by mRNA from BMDM cultured for 18 hours in TERS CM or in vehicle Veh CM with or without 4μ8C (30 μM) (n = 3) or GSK2656157 (10 nM) (n = 2). Error bars represent SEM. Data are included in S2 Data. BMDM, bone marrow–derived macrophage; CM, conditioned medium; IRE1α, inositol-requiring enzyme 1; PERK, PKR-like ER kinase; RT-qPCR, reverse transcriptase quantitative PCR; TERS CM, transmissible ER stress CM; Vegf, vascular endothelial growth factor. (PDF) [file pbio.3000687.s010.pdf]
