# Supplementary material for: IRE1α regulates macrophage polarization, PD-L1 expression, and tumor survival
Source: PLoS Biol. 2020 Jun 10;18(6):e3000687. doi: 10.1371/journal.pbio.3000687 (PMC7307794; doi:10.1371/journal.pbio.3000687)
Supplement: S5 Fig — For each mouse, genomic DNA was extracted from an ear punch and subjected to 3 PCR experiments. The first PCR (upper panel) used primers designed to evaluate the floxed status of Ern1 or Xbp1, with the floxed band appearing at 229 bp (Ern1) or 141 bp (Xbp1) and the wild-type band appearing at 254 bp (Ern1) or 183 bp (Xbp1). The band at approximately 200 bp in Ern1 is nonspecific. The second PCR (middle panel) used primers to detect the presence of the Cre insertion following the LysM promoter, with the Cre insertion appearing at approximately 700 bp. The band at 350 bp signifies the LysM promoter without Cre insertion (wild type). The third PCR (lower panel) used primers specific for the wild-type LysM promoter (without Cre), which appears 350 bp. CKO, conditional knock-out; Xbp1, X-box binding protein 1. (PDF) [file pbio.3000687.s005.pdf]

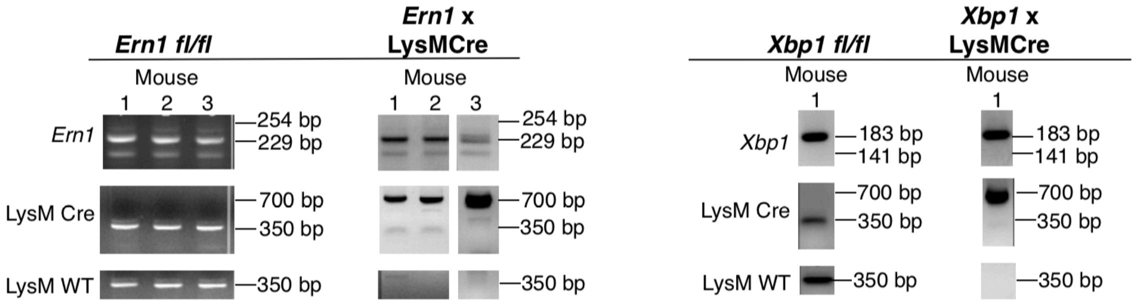

ID		Sequence
XBP1	F	5’ CAAGGTGGTTCACTGCCTGTAATG 3’
	R	5’ ACTTGCACCAACACTTGCCATTTC 3’
XBP1s	F	5’ GAACCAGGAGTTAAGAACACG 3’
	R	5’ AGGCAACAGTGTCAGAGTCC 3’
ERN1	F	5’ CCCTGCCAGGATGGTCATGG 3’
	R	5’ CCGAGCCATGAGAAACAAGG 3’
LysM WT	F	5’ CTTGGGCTGCCAGAATTTCTC 3’
	R	5’ TTACAGTCGGCCAGGCTGAC 3’
Lysm Mutant	F	5’ CTTGGGCTGCCAGAATTTCTC 3’
	R	5’ CCCAGAAATGCCAGATTACG 3’

S5 Fig
