# Supplementary material for: IRE1α regulates macrophage polarization, PD-L1 expression, and tumor survival
Source: PLoS Biol. 2020 Jun 10;18(6):e3000687. doi: 10.1371/journal.pbio.3000687 (PMC7307794; doi:10.1371/journal.pbio.3000687)
Supplement: S9 Fig — Black stands for the original gene sets. Blue and yellow colored genes are used in the aggregate pathway score after filtering out genes with less than 500 and 1,000 read counts, respectively. IRE1α, inositol-requiring enzyme 1; PERK, PKR-like ER kinase. (PDF) [file pbio.3000687.s009.pdf]

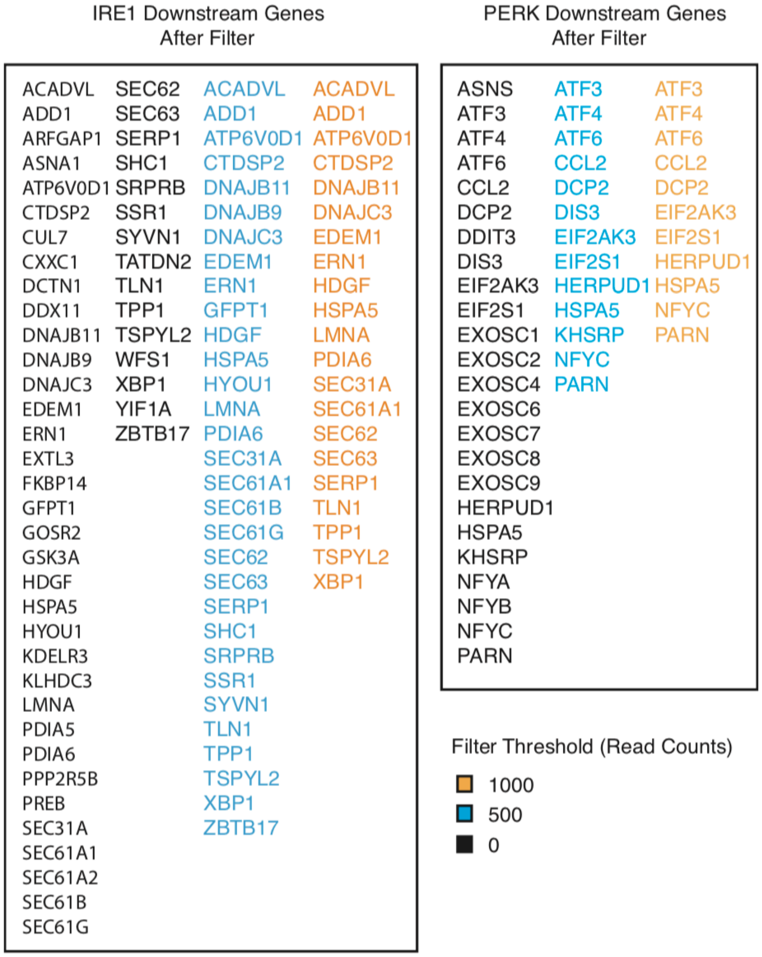
IRE1 Downstream Genes
After Filter
ACADVL ADD1 ARFGAP1 ASNA1 ATP6V0D1 CTDSP2 CUL7 CXXC1 DCTN1 DDX11 DNAJB11 DNAJB9 DNAJC3 EDEM1 ERN1 EXTL3 FKBP14 GFPT1 GOSR2 GSK3A HDGF HSPA5 HYOU1 KDELR3 KLHDC3 LMNA PDIA5 PDIA6 PPP2R5B PREB SEC31A SEC61A1 SEC61A2 SEC61B SEC61G
SEC62 SEC63 SERP1 SHC1 SRPRB SSR1 SYVN1 TATDN2 TLN1 TPP1 TSPYL2 WFS1 XBP1 YIF1A ZBTB17
ACADVL ADD1 ATP6V0D1 CTDSP2 DNAJB11 DNAJB9 DNAJC3 EDEM1 ERN1 GFPT1 HDGF HSPA5 HYOU1 LMNA PDIA6 SEC31A SEC61A1 SEC61B SEC61G SEC62 SEC63 SERP1 SHC1 SRPRB SSR1 SYVN1 TLN1 TPP1 TSPYL2 XBP1 ZBTB17
ACADVL ADD1 ATP6V0D1 CTDSP2 DNAJB11 DNAJC3 EDEM1 ERN1 HDGF HSPA5 LMNA PDIA6 SEC31A SEC61A1 SEC62 SEC63 SERP1 TLN1 TPP1 TSPYL2 XBP1
PERK Downstream Genes
After Filter
ASNS ATF3 ATF4 ATF6 CCL2 DCP2 DDIT3 DIS3 EIF2AK3 EIF2S1 EXOSC1 EXOSC2 EXOSC4 EXOSC6 EXOSC7 EXOSC8 EXOSC9 HERPUD1 HSPA5 KHSRP NFYA NFYB NFYC PARN
ATF3 ATF4 ATF6 CCL2 DCP2 DIS3 EIF2AK3 EIF2S1 HERPUD1 HSPA5 KHSRP NFYC PARN
ATF3 ATF4 ATF6 CCL2 DCP2 EIF2AK3 EIF2S1 HERPUD1 HSPA5 NFYC PARN
Filter Threshold (Read Counts)
1000
500
0

S9 Fig
