# Supplementary material for: IRE1α regulates macrophage polarization, PD-L1 expression, and tumor survival
Source: PLoS Biol. 2020 Jun 10;18(6):e3000687. doi: 10.1371/journal.pbio.3000687 (PMC7307794; doi:10.1371/journal.pbio.3000687)
Supplement: Attachment — Submitted filename: Response to reviewers.docx [file pbio.3000687.s013.docx]

**Response to reviewers**

Reviewer #1

1. *the authors should comment on the variability of the samples tested in figure 1 B to E; 3E; 5A and 5D.*

We thank the reviewer for allowing us to explain the variability in our data.

Fig. 1B. Panels B and C refer to B16.F10 tumors from tumor bearing mice (N=4) where mice were sacrificed and where we harvested two distant portions of tumor and examined them independently (technical replicates). As a baseline control we used the pooled bone marrow from two tumor bearing hosts. Panels D and E refer to adenomas from APC mice (N=5) from which we also harvested the spleen and the bone marrow, which were then processed and analyzed separately.  We agree that there exists some variability in results but this is not surprising considering that the experiments were performed using cells isolated from primary tissues. Nevertheless, variability is attributable to differences between *mice*, not the PCR assays, since values in the same mouse were consistent.

Fig. 3E. The variability observed reflects the fact that data for *Ern1* and *Xbp1* KO macrophages were collected and run at different times; each category includes two experiments, whose data were pooled to create the final panels. The results are, therefore, within acceptable ranges of inter-experiments variability.

Fig. 5A. We agree that there is variability within each group. However, we also believe that the variability is reasonable and not outside the expected range when working with primary mouse cells. Despite that, the analysis showed significance, which is what we report.

Fig. 5D. The observed variability in the *Ern1* KO macrophages is the key finding of the experiment, which we interpret as a loss of connectivity within the RIDD signature. This message is the Results section on page 14.

2. *total PERK expression is missing in Figure S3A.*

We agree with the reviewer and, in retrospect, this could have been done. However, this experiment was performed with the sole intention to assess the efficacy of the GSK inhibitor on PERK phosphorylation (which it does). We based our reasoning on a previous report showing the inhibitor does not decrease the total amount of the PERK protein but only its phosphorylation (Axten et al., *J. Med. Chem.* 2012; <https://doi.org/10.1021/jm300713s>).

3. *the authors should define the abbreviations used in Table 1.*

A new Table 1 is provided in which the TCGA acronyms have been replaced with the cancer type.

4. *the authors should correct the typos:*

*Page 8: 'Grp78 instead of Gr78'*

Corrected

*Figure 3: Chaperones Cheparones*

Using the spell-check function we could not locate the typo. We will double check the galleys when available.

5. *the authors further should comment the discrepancy between the results and in regards to PERK involvement in the PDL1 regulation. Could the data presented in Table 1 help for this?*

In the Discussion we have already pointed out that our result is at odd with a recent report in a murine cancer cell. While we do not see how Table 1 can help resolve this conundrum. However, our analysis of bulk tumor finds higher correlation between ERN1 and CD274 in the high M0 infiltration group, but higher correlation between EIF2AK3 and low M0 infiltration. This is in line with the suggestion that EIF2AK3 is more of a determinant of

CD274 expression in non M0 cells, whereas ERN1 is more of a determinant in the macrophages.

The conundrum is interesting from a therapeutic perspective since as mentioned in the paper there is growing evidence that it is PD-L1 on myeloid cells, not on tumor cells, that predicts the response to immune checkpoint inhibitors.

In the text we have added language to the effect that the difference may likely reflect difference in regulation of this gene and gene product in different cell types. Page 23: line 13-15.

Reviewer #2

1. *add a bit more detail on the potential of IRE1 inhibition in TIMs e.g which tumors, and under which circumstances? the role of IRE1-XBP1 in immune cells in the TME*

To this effect we added new language on page 21: line 22-24; page 22: line 1-5; page 23: line 19-20.

2. *An overactive IRE1α-XBP1 pathway may reprogram M1 macrophages toward an increased metabolic state, in conditions like Cystic Fibrosis, with increased glycolytic rates and mitochondrial function, and exaggerated production of TNF and IL-6.*

To this effect we added we added new language on page 21: line 11-15.
